# The Effect of Comorbidity on Glycemic Control and Systolic Blood Pressure in Type 2 Diabetes: A Cohort Study with 5 Year Follow-Up in Primary Care

**DOI:** 10.1371/journal.pone.0138662

**Published:** 2015-10-01

**Authors:** Hilde Luijks, Marion Biermans, Hans Bor, Chris van Weel, Toine Lagro-Janssen, Wim de Grauw, Tjard Schermer

**Affiliations:** 1 Department of Primary and Community Care, Radboud university medical center, Nijmegen, The Netherlands; 2 Australian Primary Health Care Research Institute, Australian National University, Canberra, Australia; Heinrich-Heine University, Faculty of Medicine, GERMANY

## Abstract

**Aims:**

To explore the longitudinal effect of chronic comorbid diseases on glycemic control (HbA_1_C) and systolic blood pressure (SBP) in type 2 diabetes patients.

**Methods:**

In a representative primary care cohort of patients with newly diagnosed type 2 diabetes in The Netherlands (*n* = 610), we tested differences in the five year trend of HbA_1_C and SBP according to comorbidity profiles. In a mixed model analysis technique we corrected for relevant covariates. Influence of comorbidity (a chronic disease already present when diabetes was diagnosed) was tested as total number of comorbid diseases, and as presence of specific disease groups, i.e. cardiovascular, mental, and musculoskeletal disease, malignancies, and COPD. In subgroup effect analyses we tested if potential differences were modified by age, sex, socioeconomic status, and BMI.

**Results:**

The number of comorbid diseases significantly influenced the SBP trend, with highest values after five years for diabetes patients without comorbidity (p = 0.005). The number of diseases did not influence the HbA_1_C trend (p = 0.075). Comorbid musculoskeletal disease resulted in lower HbA_1_C at the time of diabetes diagnosis, but in higher values after five years (p = 0.044). Patients with cardiovascular diseases had sustained elevated levels of SBP (p = 0.014). Effect modification by socioeconomic status was observed in some comorbidity subgroups.

**Conclusions:**

Presence of comorbidity in type 2 diabetes patients affected the long-term course of HbA_1_C and SBP in this primary care cohort. Numbers and types of comorbidity showed differential effects: not the simple sum of diseases, but specific types of comorbid disease had a negative influence on long-term diabetes control parameters. The complex interactions between comorbidity, diabetes control and effect modifiers require further investigation and may help to personalize treatment goals.

## Introduction

Important reasons to achieve good diabetes control are to prevent (progression of) diabetes-related complications and occurrence of cardiovascular disease [1,2]. However, diabetes patients with extensive comorbidity may benefit less from intensive blood glucose control, which was associated with reduced 5-year incidence of cardiovascular events in an observational study, but not in patients with high comorbidity scores [3]. Comorbidity, the co-occurrence of other medical conditions in addition to a specific index disease such as diabetes [4,5], is a prevalent phenomenon among diabetes patients [6–10]. More than 70% have at least one chronic non-cardiovascular disease when diabetes is diagnosed [7]. Comorbidity is related to unfavorable outcomes in terms of quality of life and health care utilization [11–14].

Knowledge of the impact of patient characteristics such as sex [15] socio-economic status (SES) [16] and body mass index (BMI) [17] on the prognosis of diabetes helps in making individualized diabetes treatment plans, and its importance is increasingly recognized. Comorbidity can be regarded as yet another patient characteristic that needs to be accounted for when formulating individualized diabetes treatment targets [2,18]. However, specific recommendations on how to take these important characteristics into account in daily practice are scarce [19]. Studies quantifying the effect of comorbidity on diabetes control in type 2 diabetes reported inconsistent findings, describing beneficial, negative, and no effects of comorbidity on diabetes control [20–24]. These studies had several limitations: they looked at a small or unclear selection of comorbid diseases only, they generally had follow-up periods of six months or less, and looked at study samples that were not representative for the overall population of patients with diabetes. These factors may contribute to differences in the results found. Particular disease combinations have received more interest in the literature, for example diabetes and depression, although the direction in their relationship and any association with diabetes outcomes remain unclear [25]. This stresses the importance to investigate the impact of comorbidity on long-term diabetes outcomes in representative samples of diabetes patients, with close monitoring of diabetes control and comprehensive recording of comorbidity. More knowledge of the types of comorbidity associated with diabetes control in ‘real life daily practice’ could help clinicians in further developing diabetes management, in which treatment goals better account for individual patients’ comorbidity profiles.

The aim of this observational study was to explore the long-term longitudinal effects of chronic comorbid disease(s) on glycemic control and systolic blood pressure (SBP) in an unselected primary care cohort of patients with type 2 diabetes receiving care as usual. Our primary interest was in the effect of patients’ number of comorbid diseases, secondary interest in the effect of specific types of comorbid disease. We did not exclude any type of chronic comorbid disease to be studied in advance. We distinguished comorbid diseases that are either related or unrelated to diabetes and explored the effects in different subgroups.

## Materials and Methods

### Design and study subjects

We used data from a dynamic cohort of diabetes patients registered in the Continuous Morbidity Registration (CMR), a family practice registration network in the Nijmegen region, the Netherlands. These four CMR practices have been recording all morbidity that is presented to the family physician (FP) on a daily basis from 1967 onwards [26]. The database reflects the health care system in the Netherlands [27] where patients are registered with a family practice and access all healthcare through that practice. FPs have an overview of all health problems of their patients. Details on the composition of our dynamic diabetes cohort are described elsewhere [7]. In short, we included all adult patients (≥18 years of age) with a new diagnosis of type 2 diabetes within the study’s observation period (1 January 1985 to 31 December 2006). The observation time for individual patients began with the start of the study period (1 January 1985), also capturing the data from patients who had already been registered in a CMR practice before 1985, or the date of a patient’s enrolment as a patient in a CMR practice, whichever occurred first. The observation period ended either at the end of our study period (31 December 2006), or with a patient’s death or deregistration from the practice, whichever occurred first. The CMR contains each patient’s date of birth, gender and socioeconomic status (SES), based on the Dutch Standard Classification of Occupations [28] classified as low, moderate, or high [29].

As part of the Nijmegen Monitoring Project (NMP), founded in 1985, the four CMR practices have also been participating in the systematic recording of diagnostic and monitoring measurements of diabetes and hypertension [30]. The NMP database includes demographic data, relevant medical history, physical diagnostics (e.g. blood pressure, weight, height), and laboratory data (e.g. HbA_1_C, glucose levels). Monitoring data are collected by the FPs and practice nurses during routine diabetes check-up visits 4 times per year for all diabetes patients under FP care. Since 1992, once a year a more extensive control visit includes screening for complications of diabetes and hypertension (i.e. retinopathy, nephropathy, and risk of diabetic foot ulceration). The family practices involved have shown to provide good quality diabetes care [30].

For the current study we used data from the four practices who provide data to both the CMR and the NMP database. The CMR database provided the comorbidity data and the NMP database the diabetes control and outcome data for the same patients. Patients did not specifically consent for use of their medical data concerning the current study. All GPs gave permission to extract data from the electronic medical records for research purposes and informed their patients, who could object to the use of their data. Those who opt-out for data extraction for research purposes continue to receive care as usual. Data are collected for observational studies and extraction from the medical records occurs de-identified. The CMR and NMP registries comply with the Code of Conduct for Health Research, which has been approved by the Dutch Data Protection Authorities (*College Bescherming Persoonsgegevens*, *CBP*) for conformity with the applicable Dutch privacy legislation, and are congruent with the Declaration of Helsinki. For this study, approval of an external ethics committee was not required.

### Outcome measures

We assessed the longitudinal development of the variables of interest: HbA_1_C (in %; DCCT aligned–the current unit during the study period), and SBP (in mmHg). Measurement of HbA_1_C is performed at the annual check-up visits and samples are analyzed in certified laboratories [31]. Practices retained the same laboratories throughout the study period. Blood pressure measurement is performed in the family practice at every check-up visit. Data collection from the diabetes diagnosis onwards occurred as part of routine care throughout patients’ registration with the practice, thus led to longitudinal data to be collected at irregular time intervals. In order to include patients with sufficient follow-up time starting from the diagnosis, we included patients with their first measurement performed within the first four months after the diabetes diagnosis, and labeled these as baseline measurements. All subsequent measurements were also included. Patients with the first measurement more than four months after the diabetes diagnosis were excluded from further analyses.

### Comorbidity

The CMR enabled us to distinguish an extensive range of comorbid diseases. Details on the recording of comorbidity are described elsewhere [7]. In short, we considered any chronic disease as comorbidity. Only chronic diseases present at the time of the diabetes diagnosis (‘at baseline’) were included. We classified comorbid diseases into disease clusters, in accordance with the International Classification of Primary Care (ICPC)-1 [32]. Consequently, the effect of comorbidity could be analyzed for single diseases, for patients’ total number of comorbid diseases, and for clusters of diseases. The number of comorbid diseases was the main focus of this study. In the analysis of specific types of comorbidity we were particularly interested in comorbidity that is expected to interact with diabetes management in various ways: diseases that may influence FPs’ and patients’ priority setting regarding the healthcare provided, or patients’ opportunities to adhere to lifestyle advices, and also diseases with either comparable-or conflicting- management plans. Therefore we distinguished ‘concordant’ comorbidity: diseases with similar pathogenesis and disease management plans as diabetes (cardiovascular diseases in the current study) [7,33]. All other types of comorbidity were regarded as ‘discordant’ comorbidity (diseases unrelated to diabetes). For our longitudinal analysis we selected comorbid malignancies, cardiovascular, mental, and musculoskeletal diseases as disease clusters, and comorbid COPD as a single disease of particular interest. We refer to these selected conditions as ‘selected comorbidity’ from here onwards. S1 Appendix: Classification of comorbidity provides further details on the classification of disease clusters included in this study.

Because the CMR database contains longitudinal data we were able to distinguish prevalent comorbidity in a particular patient at the date of the diabetes diagnosis, and incident comorbidity after the diabetes diagnosis. For the current study we only analyzed the effect of existing (prevalent) comorbid diseases on diabetes outcomes over time. The CMR’s internal quality control and the extensive experience in morbidity recording before the start of our observation period ensured optimal consistency in diagnoses throughout our cohort [26].

This paper describes the main results from a larger research project, studying the effects of a number of comorbid diseases on long-term diabetes control parameters. Description of the effects of some specific types of comorbidity on diabetes control parameters in complex interaction models fell beyond the scope of the current paper and will be reported separately [34].

### Statistical analysis

SPSS (version 20.0) and SAS (version 9.02) supported the analyses. We used descriptive statistics to provide characteristics of the study population and comorbidity profiles at baseline. Differences in outcomes between male and female patients and between patients from different socioeconomic classes were compared using Chi-square tests and independent t-tests for categorical and continuous variables, respectively.

To address our research question we explored linear trends for both HbA_1_C and SBP in the five years after the diabetes diagnosis for patients with different baseline comorbidity profiles.

First we tested if the number of comorbid diseases at baseline (categorized as 0; 1 or 2; or ≥3) influenced the HbA_1_C and SBP trend. We applied a random intercept mixed model analysis using measurements nested within patients [35]. All measurements within the first five years after the diabetes diagnosis contributed to this mixed model. With the same approach we tested the potential influence of the presence of selected comorbidity. We added an interaction term ‘time’ by ‘comorbidity’ (total number, or type of comorbidity) to the model to explore differences in the HbA_1_C and SBP trends according to comorbidity. Separate analyses were performed for different types of selected comorbidity. In these comparisons of diabetes patients with different comorbidity profiles, we entered sex, age at diabetes diagnosis, SES, and BMI (handled as ‘last observation carried forward’ [35]) as potential confounders. Values for age and BMI were handled as continuous variables in the mixed model, but we categorised them as ‘low’, ‘intermediate’, and ‘high’ values to facilitate (graphical) presentation of the results. The categorisation was based on the limits of the first, second (i.e., the median) and third quartile of the distribution of age and BMI values of the patients who contributed to the analysis. When exploring the effect of selected comorbidity (e.g., malignancy) on the five year HbA_1_C and SBP trend, ‘presence of other selected comorbidity’ was also entered as potential confounder.

Second, we performed subgroup effect analyses, to explore if potential differences in the trend of HbA_1_C and SBP, according to the number of comorbid diseases, were modified by sex, age, SES, or BMI. The confounders in the initial analysis were now tested for potential effect modification separately by adding an interaction term ‘time’ by ‘comorbidity’ by ‘potential effect modifier’ (e.g., ‘sex’) to the model. Non-significant terms were removed in a stepwise hierarchical backward elimination procedure [35]. In the cases where no significant results arose from the subgroup effect analysis, the first model (without subgroup effect analysis) defined the results.

P-values <0.05 were considered statistically significant.

### Sensitivity analysis

Since our observation period covered a lengthy time frame, we performed a sensitivity analysis to test if the calendar period in which patients’ diabetes was diagnosed, influenced the findings. Diabetes diagnosis calendar period was categorized, corresponding to the prevailing diabetes guidelines (i.e., 1985–1989, 1990–1999, 2000–2006), as published by the Dutch College of General Practitioner’s (publication of first, second, and third version in 1989, 1999, and 2006 respectively) [36]. We performed a subgroup analysis including this variable ‘calendar period’.

## Results

### Study subjects and baseline characteristics

**Fig 1:**
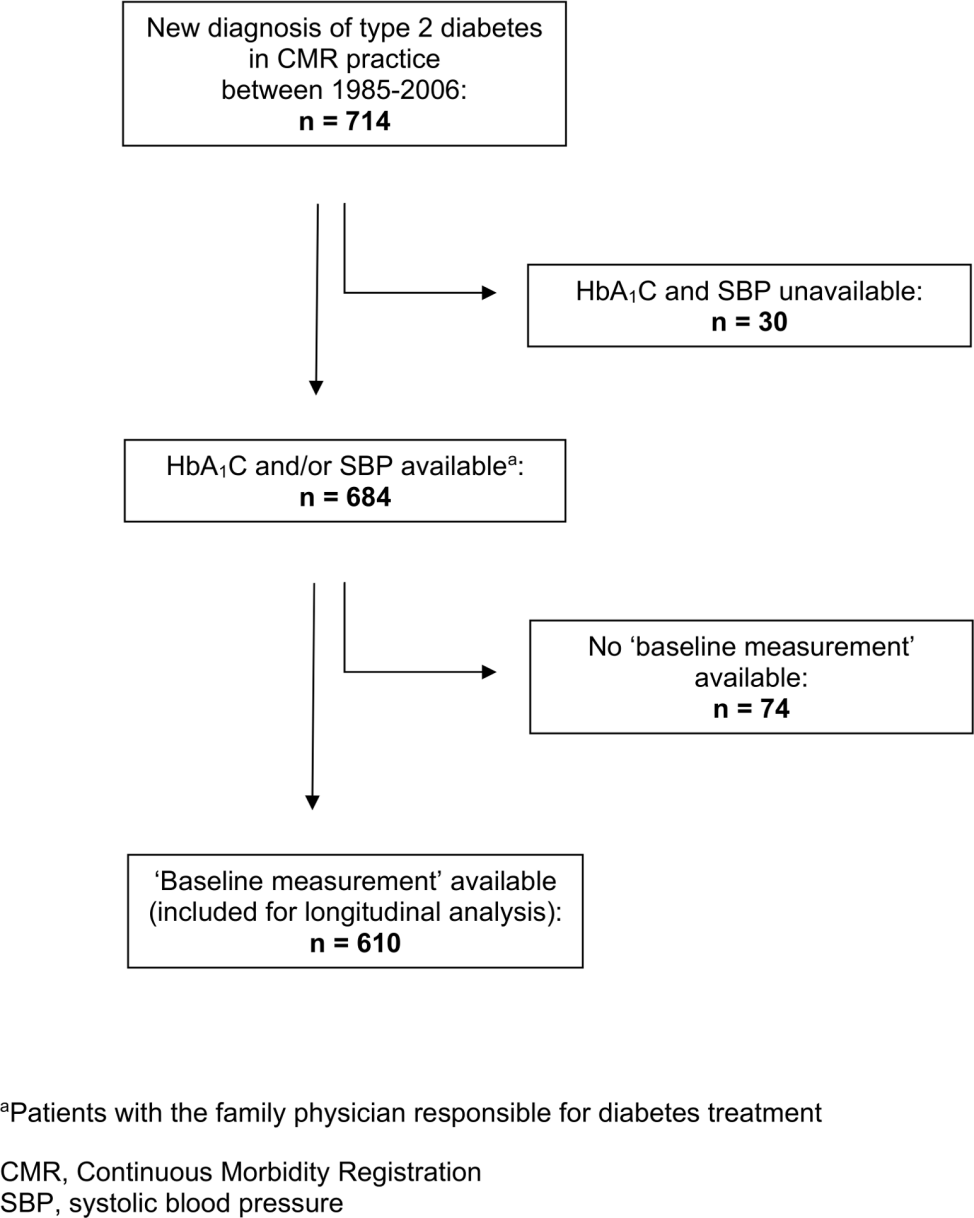
Flow chart of patient selection from the CMR general practice database.

We identified 714 patients with a new diagnosis of type 2 diabetes within the study period (1985–2006). Outcome measurements were available in 684 patients. Of these, 610 patients had a first measurement of HbA_1_C and/or SBP within four months from diagnosis and were included for longitudinal analysis. Fig 1 shows a flow chart of our study population.

Mean age at diabetes diagnosis was 63.0 (SD 12.5) years. 48.2% of the patients included were males. For some comorbid diseases the baseline prevalence differed between males and females and by SES class. Table 1 shows the baseline characteristics for the total sample, and by sex and SES separately. For both sexes the mean HbA_1_C at baseline was 7.4% (SD 1.7% for males, 2.0% for females). Mean SBP at baseline was 147.4 (SD 19.4) for males, 153.8 (SD 20.3) for females.

**Table 1:** Baseline patient characteristics, according to sex and SES.

	Total included	Males	Females	P-value^a^	Low SES	Middle SES	High SES	P-value^b^
Variables	(n = 610)	(n = 294)	(n = 316)		(n = 315)	(n = 242)	(n = 48)	
***Patient characteristics***								
**SES** ^**b**^, n (%)								
Low	315 (52.1)	145 (49.7)	170 (54.3)	.45				x
Middle	242 (40.0)	121 (41.4)	121 (38.7)		x	x	x	
High	48 (7.9)	26 (8.9)	22 (7.0)					
**End of follow-up, reason**, n (%)								
End of study period	396 (64.9)	193 (65.6)	203 (64.2)	.85	202 (64.1)	158 (65.3)	33 (68.8)	.69
Deceased	128 (21.0)	62 (21.1)	66 (20.9)		64 (20.3)	55 (22.7)	8 (16.7)	
Moved / left practice	86 (14.1)	39 (13.3)	47 (14.9)		49 (15.6)	29 (12.0)	7 (14.6)	
**Year of diabetes diagnosis**, n (%)								
1985–1989	83 (13.6)	38 (12.9)	45 (14.2)	.32	39 (12.4)	40 (16.5)	4 (8.3)	.11
1990–1999	235 (38.5)	106 (36.1)	129 (40.8)		135 (42.9)	83 (34.3)	16 (33.3)	
2000–2006	292 (47.9)	150 (51.0)	142 (44.9)		141 (44.8)	119 (49.2)	28 (58.3)	
**Age at diabetes diagnosis**, mean (SD; range), years	63.0 (12.5; 23–95)	61.9 (12.2; 24–89)	64.1 (12.7; 23–95)	.03	61.9 (12.4; 23–91)	64.3 (12.4; 24–95)	65.2 (11.7; 32–88)	.04
**Follow-up time**,mean (SD; range), years	6.2 (4.6; 0.1–21.9)	5.9 (4.4; 0.1–21.9)	6.5 (4.8; 0.2–21.2)	.32	6.3 (4.5; 0.3–21.9)	6.4 (4.9; 0.1–20.9)	5.2 (3.6; 0.2–17.0)	.27
**Measurements per patient, total number**, mean (median; SD; range)	21.8 (17.5; 18.2; 1–106)	20.1 (17; 16.3; 1–92)	23.4 (19; 19.7; 1–106)	.02	22.1 (18; 18.2; 1–106)	22.1 (17.5; 18.7; 1–96)	19.3 (16.5; 16.7; 1–95)	.59
**BMI at baseline**, mean (95%CI; SD; range), kg/m^2^	29.8 (29.4–30.2; 5.1; 18.9–54.1)	29.0 (28.5–29.6; 4.5; 18.9–47.9)	30.5 (29.9–31.2; 5.5; 20.6–54.1)	< .001	30.2 (29.6–30.8; 5.3; 19.6–52.5)	29.5 (28.8–30.1; 4.9; 18.9–54.1)	29.0 (27.7–30.4; 4.4; 20.6–43.8)	.13
***Comorbidity data***								
**Comorbid diseases present at baseline**, mean number (SD; range)	2.8 (2.3; 0–12)	2.4 (2.1; 0–9)	3.2 (2.5; 0–12)	< .001	2.9 (2.3; 0–12)	2.8 (2.3; 0–12)	2.5 (2.1; 0–9)	.62
**Cardiovascular comorbidity at baseline**, Present, n (%)	390 (63.9)	175 (59.5)	215 (68.0)	.03	193 (61.3)	167 (69.0)	29 (60.4)	.14
**Musculoskeletal comorbidity at baseline**, Present, n (%)	197 (32.3)	78 (26.5)	119 (37.7)	.003	102 (32.4)	79 (32.6)	16 (33.3)	.99
**Mental comorbidity at baseline**, Present, n (%)	140 (23.0)	46 (15.6)	94 (29.7)	< .001	85 (27.0)	49 (20.2)	5 (10.4)	.02
**Comorbid malignancy at baseline**, Present, n (%)	42 (6.9)	21 (7.1)	21 (6.6)	.80	25 (7.9)	13 (5.4)	4 (8.3)	.46
**Comorbid COPD at baseline**, Present, n (%)	63 (10.3)	34 (11.6)	29 (9.2)	.33	37 (11.7)	24 (9.9)	2 (4.2)	.26
**No comorbidity at baseline** (0 diseases), n (%)	96 (15.7)	57 (19.4)	39 (12.3)	.02	50 (15.9)	37 (15.3)	6 (12.5)	.83
**Cardiovascular comorbidity only at baseline,** n (%)	88 (14.4)	49 (16.7)	39 (12.3)	.13	39 (12.4)	39 (16.1)	9 (18.8)	.31
**Discordant comorbidity only at baseline**, n (%)	124 (20.3)	62 (21.1)	62 (19.6)	.65	72 (22.9)	38 (15.7)	13 (27.1)	.06
**Both concordant and discordant comorbidity at baseline**, n (%)	302 (49.5)	126 (42.9)	176 (55.7)	.002	154 (48.9)	128 (52.9)	20 (41.7)	.32
**Mental comorbidity only at baseline,** n (%)	8 (1.3)	3 (1.0)	5 (1.6)	.54	5 (1.6)	1 (0.4)	1 (2.1)	.36
**Musculoskeletal comorbidity only at baseline,** n (%)	17 (2.8)	8 (2.7)	9 (2.8)	.92	10 (3.2)	5 (2.1)	2 (4.2)	.62
**Comorbid malignancy only at baseline,** n (%)	4 (0.7)	3 (1.0)	1 (0.3)	.28	2 (0.6)	1 (0.4)	1 (2.1)	.43
**Comorbid COPD only at baseline,** n (%)	4 (0.7)	3 (1.0)	1 (0.3)	.28	3 (1.0)	1 (0.4)	0 (-)	.62

^a^ P-value for difference between male and female values.

^b^ P-value for difference between low, middle, and high class of SES. Number of measurements available for SES: 605 (missing: n = 5). SES, socio-economic class.

Patients without baseline measurements (n = 104, Fig 1) tended to be more often male and to have higher SES, higher age, and more comorbid diseases (including more often malignancy) at the time of their diabetes diagnosis, compared to those with baseline measurements available. None of these differences reached statistical significance.

### Influence of comorbidity on the HbA_1_C and SBP trends

HbA_1_C at time of diabetes diagnosis tended to be lower when patients had a higher number of comorbid diseases at baseline, but the number of comorbid diseases at baseline did not significantly influence the longitudinal development of HbA_1_C (p = 0.075). After five years, patients without baseline comorbidity had worst glycemic control. The number of comorbid diseases at baseline significantly influenced the development of SBP over time (p = 0.005). Absolute differences in mmHg were small. Patients without comorbid disease at baseline showed to have highest SBP after five years.

For the selected comorbidity we observed a different time trend of HbA_1_C for patients with *versus* without musculoskeletal disease at baseline (p = 0.044). Those with musculoskeletal disease started with lower HbA_1_C values but had higher values after five years. Cardiovascular comorbidity significantly affected the longitudinal development of SBP (p = 0.014), resulting in higher SBP values from the diabetes diagnosis onwards. No statistically significant effects were observed for the other types of selected comorbidity.

**Fig 2:**
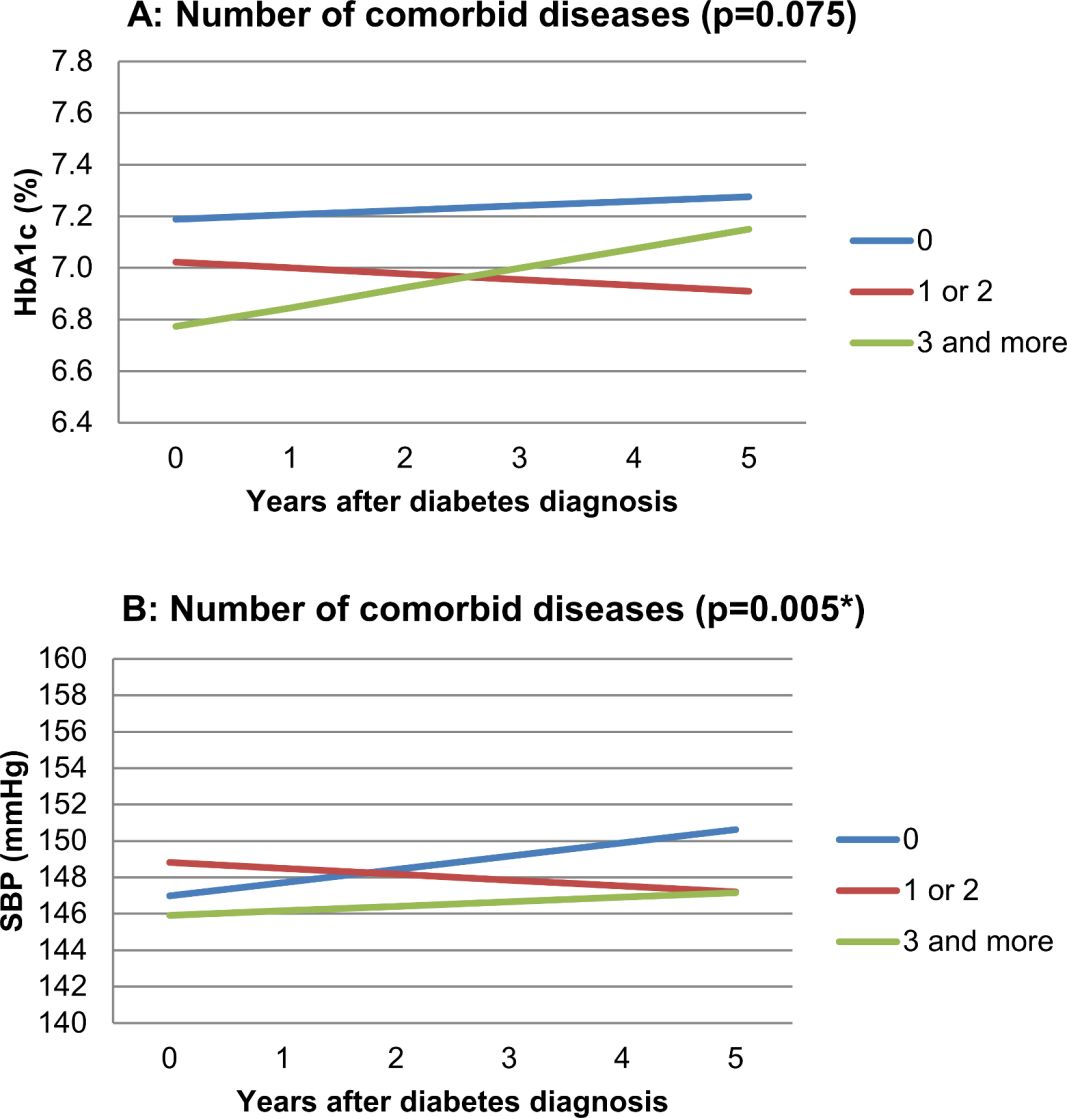
Effect of number of comorbid diseases on five year HbA_1_C trend (Panel A, p 0.075) and on five year SBP trend (Panel B, p 0.005*). The reference category is male sex, low SES, median age, median BMI. Beta-coefficients (slopes for graph lines): Panel A (% HbA_1_C per year): 0 diseases: +0.0175; 1–2 diseases: -0,0225; ≥3 diseases: +0,0714. Panel B (mmHg per year): 0 diseases: +0,728; 1–2 diseases: -0,324; ≥3 diseases: +0,249. Abbreviations: BMI, Body Mass Index. SBP, systolic blood pressure. SES, socio-economic status.

**Fig 3:**
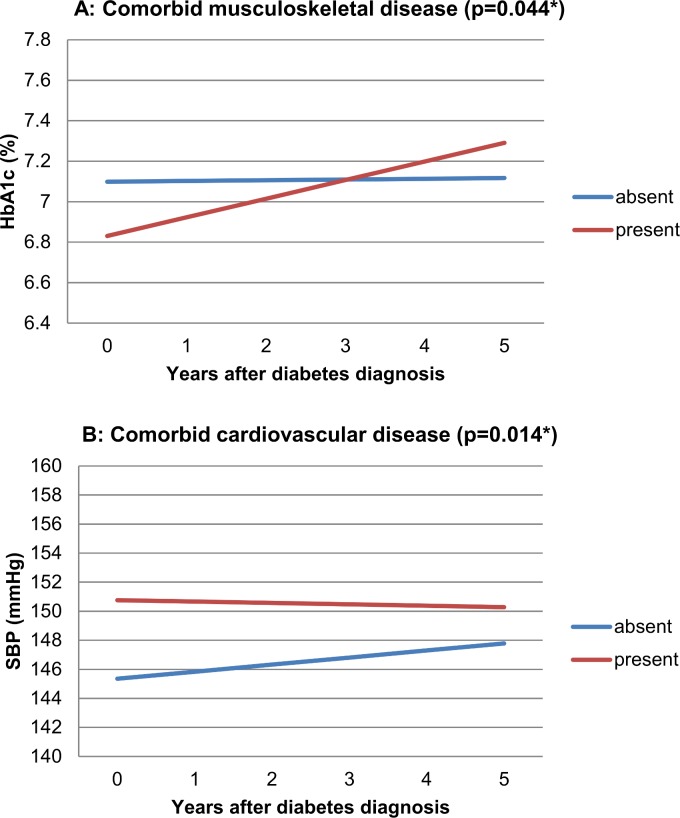
Effect of comorbid musculoskeletal disease on five year HbA_1_C trend (Panel A, p 0.044*) and of comorbid cardiovascular disease on five year SBP trend (Panel B, p 0.014*). The reference category is male sex, low SES, median age, median BMI, and ‘absence of other comorbidity’. Beta-coefficients (slopes for graph lines): Panel A (% HbA_1_C per year): Musculoskeletal disease absent: +0,0037; musculoskeletal disease present: +0,0921. Panel B (mmHg per year): Cardiovascular disease absent: +0,486; cardiovascular disease present: -0,096.

**Fig 4:**
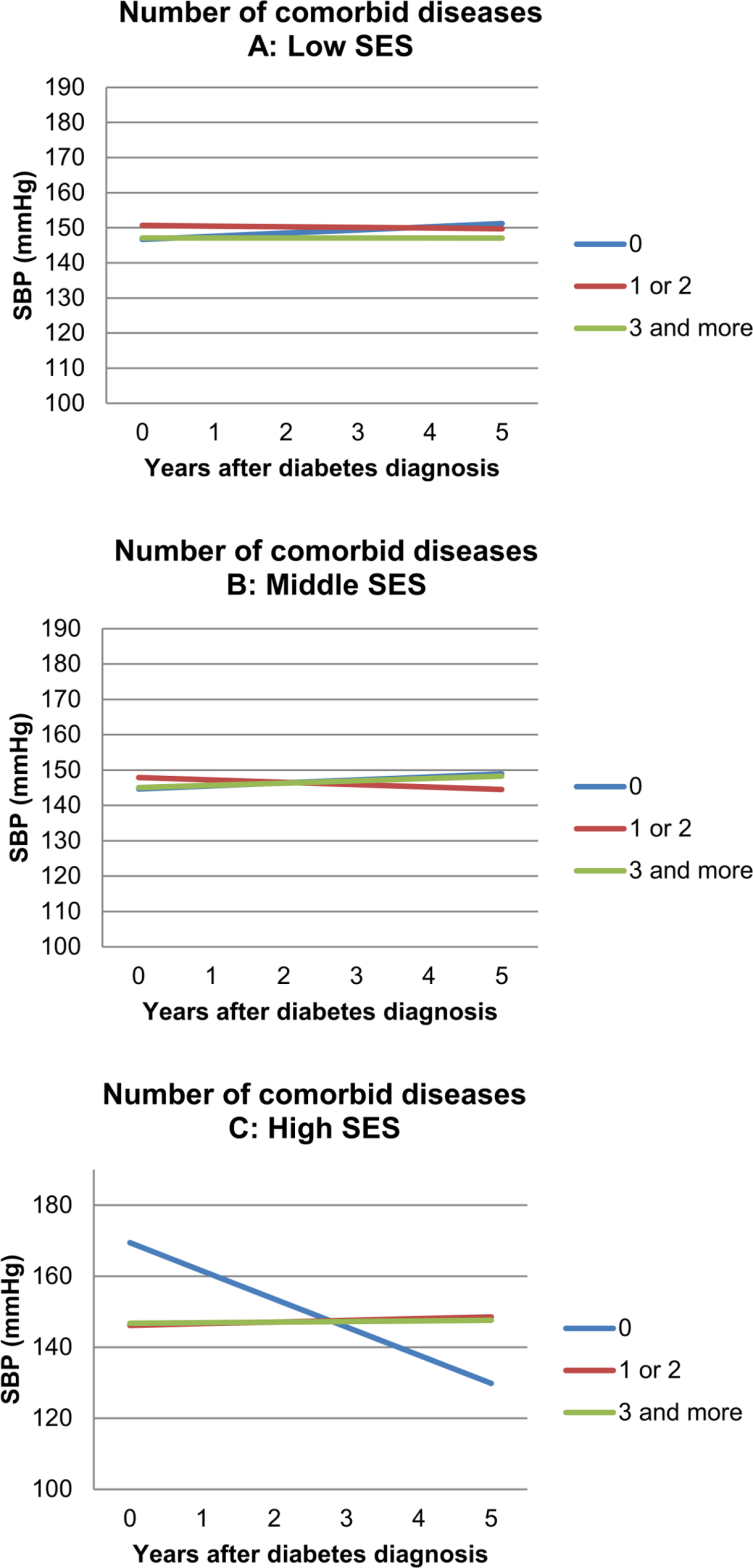
Subgroup effect analysis: Effect of number of comorbid diseases on five year SBP trend: modified by SES (p < 0.001*). **A, low SES. B, Middle SES. C, high SES.** Graphs are shown for median age and median BMI. Other variables are specified. Beta-coefficients (slopes for graph lines): Panel A (low SES; mmHg per year): 0 diseases: +0,902; 1–2 diseases: -0,193; ≥3 diseases: -0,008. Panel B (middle SES; mmHg per year): 0 diseases: +0,855; 1–2 diseases: -0,677; ≥3 diseases: +0,648. Panel C (high SES; mmHg per year): 0 diseases: -7,922; 1–2 diseases: +0,479; ≥3 diseases: +0,167.

Figs 2 and 3 present the direction of effects graphically. The lines represent the predicted values for HbA_1_C or SBP over five years from the mixed models. Corresponding p-values indicate the statistical significance of the difference between their slopes. Absence and presence of comorbid disease are determined on the date of the diabetes diagnosis. We define the (theoretical) combination of specific patient characteristics (e.g., sex, age, SES) as ‘reference category’. In the graphic presentation, graph lines represent HbA_1_C or SBP trends for subjects from this ‘reference category’. The corresponding additional effects tables (Tables 2 and 3) contain information needed to construct lines of predicted outcomes, based on the mixed model results, for other subjects than the ‘reference category’. It shows the additional effects (to be added to the graphs) for other covariates included in the model. These values are *not time dependent* and apply to *any of the comorbidity groups* displayed in the corresponding Figure. Example: Fig 3 Panel A shows predicted HbA_1_C time trends for patients with and without comorbid musculoskeletal disease (mixed model results). The reference category for these graph lines includes male sex. Table 3 (with additional effects for Fig 3, Panel A) shows an additional effect of +0.04 (% HbA_1_C) for female sex. This means 0.04 should be added to the blue line for female patients without musculoskeletal disease and 0.04 should be added to the red line for female patients with musculoskeletal disease. The p-value of 0.69 shows that this additional effect of sex on HbA_1_C in this analysis is not statistically significant. For Figs 2–4, the number of patients with complete contribution up to and including a specific year after the diabetes diagnosis (follow-up ≥ *x* years) was as follows: after 0 years: 610, after 1 year: 554, after 2 years: 484, after 3 years: 430, after 4 years: 379, after 5 years: 342. Based on the distribution of age and BMI values of the patients who contributed to the analysis, limits for ‘low’, ‘intermediate’, and ‘high’ values of age were 54, 64 and 72 years, and for BMI, these were 26.0, 28.5 and 31.8 kg/m^2^. High BMI was associated with increased HbA_1_C and SBP values and higher age with increased SBP values. In the analysis of the effect of selected comorbidity we corrected for presence of other selected comorbidity and found, for instance, that baseline cardiovascular disease increased SBP, and comorbid mental disease decreased both HbA_1_C and SBP.

**Table 2:** Additional effects for Fig 2.

Variable	Effect on HbA_1_C (%), i.e. applies to Panel A	P-value	Effect on SBP (mmHg), i.e. applies to Panel B	P-value
BMI: low	-0.07	0.004*	-2.4	< 0.001*
BMI: high	+0.10		+3.3	
Age: low	+0.07	0.17	-5.7	< 0.001*
Age: high	-0.06		+4.5	
Sex: female	-0.01	0.90	+1.9	0.13
SES: middle	-0.09	0.72	-1.9	0.27
SES: high	-0.03		+0.7	

*P-values < 0.05.

**Table 3:** Additional effects for Fig 3.

Variable	Effect on HbA_1_C (%), i.e. applies to Panel A	P-value	Effect on SBP (mmHg), i.e. applies to Panel B	P-value
BMI: low	-0.07	< 0.005*	-2.3	< 0.001*
BMI: high	+0.09		+3.1	
Sex: female	+0.04	0.69	+2.0	0.11
SES: middle	-0.08	0.77	-2.4	0.15
SES: high	-0.06		+0.1	
Age: low	+0.09	0.09	-5.0	< 0.001*
Age: high	-0.07		+4.0	
Mental disease	-0.30	0.01*	-3.5	0.02*
Cardiovascular disease	-0.10	0.37	N.A.	
Malignancy	-0.07	0.73	-4.1	0.10
COPD	0.18	0.30	-0.3	0.08
Musculoskeletal disease	N.A.		-0.2	0.84

*P-values < 0.05. N.A., not applicable.

### Subgroup effect analyses

In the subgroup effect analyses no modification was found for the effect of the number of comorbid diseases on the course of HbA_1_C. The relationship between the number of comorbid diseases at baseline and the course of SBP was significantly modified by SES (p<0.001). Since the direction of effects is not readily understood in complex interaction models, Fig 4A–4C shows the longitudinal development of SBP for subgroup differences. In the high SES group, patients without baseline comorbid diseases showed a clear decrease of SBP over time, with high baseline values and much lower values after five years. Table 4 provides information about additional effects of covariates. Note that non-significant terms were removed from the model in the hierarchical backward elimination approach.

**Table 4:** Additional effects for Fig 4 (i.e. contributing significantly to the model).

Variable	Effect on SBP (mmHg)	P-value
BMI: low	-2.5	< 0.001*
BMI: high	+3.4	
Age: low	-5.8	< 0.001*
Age: high	+4.6	

### Sensitivity analysis

No statistically significant effect modifications were found for the ‘calendar period’ subgroups on the effect of the number of comorbid diseases on HbA_1_C; or for musculoskeletal disease on HbA_1_C; or for CVD on SBP. We did observe a significant subgroup effect of ‘calendar period’ in the analysis of the number of comorbid diseases on longitudinal SBP (p = 0.023).

## Discussion

In this observational study we explored the effects of chronic comorbid diseases on the long-term longitudinal development of HbA_1_C and SBP in newly diagnosed type 2 diabetes patients in primary care. Our results show that the number of comorbid diseases at baseline influenced longitudinal development of SBP, an effect that was modified by SES. The effect of comorbidity on the longitudinal development of HbA_1_C was limited, but present in specific types of discordant comorbidity (musculoskeletal disease). Concordant cardiovascular comorbidity negatively impacted on the longitudinal SBP development. These results indicate that comorbid diseases affect long-term diabetes control parameters, with distinct patterns for different numbers and types of comorbidity, and modification of some patterns by SES. The specific information on the relation between comorbidity and diabetes control parameters provided by this explorative study needs replication first, but has the potential to offer new opportunities to deliver more personalized diabetes treatment, by taking specific types of comorbidity into account that may require different therapeutic approaches.

Our longitudinal data cover a lengthy study period, which is a major strength of this work. By deliberately including patients of all ages and with any type of comorbidity, our data are representative for the diabetes population in primary care. The data have their origin in an experienced family practice registration network and practices provide good quality diabetes care. The diabetes control parameters we used (HbA_1_C and SBP) are relevant when studying long-term diabetes control. Surrogate outcomes such as ‘intensification of medication treatment’ reflect mainly physicians’ actions, not patients’ responsiveness, and do not necessarily result in better diabetes control [22–24]. Since we only included newly diagnosed diabetes patients, comparison of diabetes control parameters over time was meaningful.

During this study period, diagnosis and treatment of some diseases studied may have changed, which could influence our findings–although it is not obvious if this would alter our findings in a specific direction, or if this would only be the case for specific types of comorbidity. This type of limitation is inherent to analyzing longitudinal data with an extensive follow-up period. In a sensitivity analysis, only the association between the number of comorbid diseases and SBP trend was significantly affected by the calendar period of the diabetes diagnosis. Exploration of this association suggested that this resulted from altered treatment of diabetes itself throughout our study period–not changes in categorizing or managing ‘comorbidity’. After all, monthly meetings ensured maximum consistency in CMR’s diagnostic labels, which are known for their high validity [37,38].

The extensiveness of our data did not allow for detailed elaboration on complex subgroup effect analyses results for various types of comorbid diseases within the current paper. Although we had smaller numbers of repeated measurements available for HbA_1_C than for SBP, in further subgroup effect analyses we observed that comorbid malignancy (the smallest group of selected comorbidity) had significant effects on the HbA_1_C trend of diabetes patients, with modification by some effect modifiers (to be reported separately). This makes lack of statistical power an unlikely explanation for the limited effects of comorbidity on HbA_1_C development observed in this study.

Among the 30 patients in our cohort without outcome data, some had not been included in the NMP registration due to extensive multimorbidity. These patients should be regarded as seriously ill patients with comorbid diabetes, in whom low priority was given to the diabetes monitoring. Some other patients without outcome measurements had their diagnosis of diabetes assigned towards the end of our study period, allowing insufficient time for the first outcome measurement registration to occur in the NMP database within the study period. Any potential bias here is probably small, since no statistically significant differences in baseline characteristics were observed between patients with and without baseline measurements. The higher proportion of diabetes diagnoses made at the end of our study period may be caused by increased attention to early detection of diabetes and by targeted screening for diabetes in the same period [39]. Due to the extensive length of the study period this did not result in insufficient long-term follow-up data.

Inclusion of lipids as diabetes control parameter would have provided added value to this study. However, after the first recognition of the importance of lipid regulation in diabetes [40]. the revised version of the Dutch College of General Practitioners diabetes guideline in 1999 resulted in increased attention to the role of lipids halfway our study period. We decided to look at one glycemic and one major cardiovascular risk outcome measure only—which already resulted in an extensive dataset.

The longitudinal outcomes are obviously influenced by prescribed medication and non-pharmacological interventions for the treatment of diabetes and comorbid diseases. In this dynamic cohort study, it was not possible to compare pharmacotherapy and lifestyle interventions longitudinally between diabetes patients with and without specific types of comorbidity. Therefore, we cannot tell if and how potential differences in therapeutic regimes may have contributed to the outcomes observed. However, such a comparison fell beyond the scope of this observational, explorative study, in which we aimed to explore the long-term associations between comorbidity and diabetes control parameters in patients receiving care as usual. The presence of comorbidity may influence FPs’ perception of benefits and feasibility of therapeutic regimes [41,42]. Specific types of prescriptions may be less appropriate or less safe in patients with particular types of comorbidity, which may influence the diabetes treatment options for these patient groups. Future longitudinal research should pay attention to the role of medication prescriptions in the association between diabetes control parameters and comorbidity. Prescription data may be handled as a potential confounder, or even as an outcome measure.

We used a well-documented, comprehensive definition of comorbidity to describe the effect of comorbidity on trends of diabetes control parameters in diabetes patients. The study design enabled accurate distinction between ‘no’, ‘some’ (one or two) and ‘many’ (three and more) comorbid diseases. This contrasts with other studies in which comorbidity could be either present or absent in a limited selection of diseases, where absence of selected comorbidity does not exclude presence of unselected types of comorbidity [21–23]. We made a clinically important distinction between related (concordant) comorbidity and unrelated (discordant) comorbidity [33]. The slightly better long-term outcomes for SBP and HbA_1_C for patients with ‘much’ comorbidity (≥3 diseases at baseline) compared to patients without comorbidity (0 diseases) are new findings that need further investigation. Different types of comorbidity appeared to affect diabetes outcomes in different ways.

The mixed model analysis technique we used was the most appropriate model to address our research question, and enabled us to optimally use the available longitudinal data. Absolute values of HbA_1_C and SBP are difficult to interpret in this study since the mixed model reports predicted values for specific (reference) groups. We deliberately formulated a broad research question. Our results should be regarded as a starting point for further research, hence care must be taken not to ‘over-interpret’ the results before they are confirmed in larger cohorts with deeper investigation of prominent associations found.

Little is known about the long-term ‘natural development’ of HbA_1_C and SBP in type 2 diabetes patients from the diabetes onset onwards. Best estimations of this ‘natural course’ probably come from control groups of large diabetes trials, of which only UKPDS [43] included newly diagnosed patients, but they excluded patients with ‘serious illness’. Our findings, showing different longitudinal outcomes according to the absence or presence of (particular types of) comorbidity, add knowledge on the ‘natural development’ of diabetes outcomes from diabetes diagnosis onwards, and especially how they are influenced by comorbidity and other effect modifiers. In other words, it showed that long-term diabetes control parameters in patients without comorbidity (typically the patients that are included in RCTs) are not representative for the entire diabetes population. Studies that look at the effectiveness of diabetes treatment but overlook comorbidity may have seriously limited generalizability of their results.

Detection of type 2 diabetes may occur in an earlier stage of the disease if comorbidity is present. The lower baseline values shortly after the diabetes diagnosis among patients with musculoskeletal disease (for HbA_1_C) or with ‘three and more’ comorbid diseases (HbA_1_C and SBP) compared to those without comorbidity suggest existence of such patterns. It may occur for example by more frequently performed laboratory tests including glucose levels in patients who already have other chronic diseases. In the group with pre-existing musculoskeletal comorbidity, HbA_1_C increased in subsequent years, resulting in worse glycemic control after five years compared to patients without musculoskeletal disease at baseline. Impaired ability for physical exercise seems a plausible explanation for the longitudinal differences. Similarly, we assume that the consistent additional effects from comorbid mental disease (reduced HbA_1_C and SBP values) is explained by a higher contact frequency of these patients with their FP [44] resulting in additional opportunities to diagnose and treat diabetes in an early stage with slightly better outcomes. The nature of comorbid cardiovascular disease, concordant with diabetes, probably explains its augmenting effect observed for SBP. It should be noted that patients with cardiovascular disease might be diagnosed with ‘hypertension’. However, the presence of the diagnostic label ‘hypertension’ alone (without presence of other cardiovascular disease, such as myocardial infarction or CVA) did not classify as ‘cardiovascular disease’ in the longitudinal analysis for the current study, and therefore ‘hypertension’ could be diagnosed also among the patients in the group ‘without cardiovascular disease’. Diabetes guidelines recommend good SBP control in ‘all’ diabetes patients—independent from presence of (cardiovascular or non-cardiovascular) comorbidity [1,2]. In this observational study we analyzed whether presence of particular numbers or types of comorbidity was associated with the attainment of different longitudinal diabetes control parameters in diabetes patients receiving care as usual from their FP. From this objective, the impossibility to correct for use of antihypertensive medication is of minor importance since, according to current guidelines, lowering SBP if values are increased is regarded as equally important in all patients.

A large number of comorbid diseases at baseline (three and more) did not result in a less favorable course of HbA_1_C and SBP, but particular types of comorbidity did. This observation—not the simple sum of diseases, but specific types of comorbid disease influence the course of diabetes control parameters—emphasizes that the diabetes care as provided by FPs is part of general healthcare delivered to ‘whole persons’, i.e. ‘person-centered care’. Apparently, the patient-specific context intervenes, in which FPs integrate disease-specific and generic patient characteristics and treatment goals as part of diabetes-specific care.

The observed effect modification of the number of comorbid diseases on the course of SBP by SES warrants further exploration. Other studies already highlighted SES as an important patient characteristic in comorbidity [6] and in diabetes research [16] but little has been reported on its role as effect modifier as described here. Further subgroup effect analysis from our own data showed that SES also modifies the effect of long-term SBP control compared between diabetes patients with and without comorbid COPD [34]. The explorative design of the current study does not allow us to give possible explanations, such as patients’ delay (to visit a doctor) or greater ability for long term risk factor control among specific SES groups.

In conclusion, this observational study showed that presence of chronic comorbid diseases affected the longitudinal course of HbA_1_C and SBP in a representative sample of newly diagnosed type 2 diabetes patients receiving care as usual. Different numbers and types of comorbid diseases showed specific patterns of influence on these outcomes. Further investigation of the complex association between diabetes, comorbidity and effect modifiers is needed to replicate our findings and to elaborate on the consequences of specific levels of diabetes control. Our observations illustrate that future diabetes studies should take the presence of comorbidity into account, and suggest that FPs’ diabetes care requires a person-centered approach, especially when comorbidity is present.

## Supporting Information

S1 Appendix Classification of comorbidity. (DOCX)

## Abbreviations

BMI: body mass index
DM: diabetes mellitus
FP: family physician
SBP: systolic blood pressure
SES: socioeconomic status
