# Supplementary material for: The Effect of Comorbidity on Glycemic Control and Systolic Blood Pressure in Type 2 Diabetes: A Cohort Study with 5 Year Follow-Up in Primary Care
Source: PLoS One. 2015 Oct 1;10(10):e0138662. doi: 10.1371/journal.pone.0138662 (PMC4591264; doi:10.1371/journal.pone.0138662)
Supplement: S1 Appendix — (DOCX) [file pone.0138662.s001.docx]

**S1: Appendix: Classification of comorbidity**

The following clusters of chronic diseases were distinguished:

- Cardiovascular disease
- Malignancy
- Musculoskeletal disease
- Mental health disease
- Respiratory disease
- Eye and ear disease
- (Male and female) urogenital disease
- Skin disease
- Digestive system disease
- Endocrine and metabolic disease
- Neurological disease
- Blood(forming organs) and lymphatics disease
- Infectious disease
- General and unspecified disease

Each disease cluster contains several chronic diseases. Within any cluster of diseases, presence of at least one chronic disease classified in this cluster was required, in order to be counted as presence of this disease cluster in a particular patient. For each separate cluster it was defined whether or not a particular patient classified for this disease cluster.

Any single chronic disease present in any of the abovementioned clusters contributed to the total sum of comorbidity as distinguished in this study (‘number of comorbid diseases’).

This study included only chronic diseases as comorbidity. Some diseases can be regarded as invariably chronic diseases (e.g. rheumatoid arthritis, schizophrenia). Other diseases may have a chronic course in a particular patient but do not necessarily do so in all cases (e.g. gout, migraine, depression). These diseases were defined as ‘conditionally chronic’ diseases and were included as comorbidity in this study only when it had a chronic course in this particular patient. These methods have been elaborated in a previous paper of the same research group.^1^

In the current study, five selected clusters of diseases were analyzed separately, in addition to the analysis of the number of comorbid diseases. These disease clusters were cardiovascular disease, malignancy, musculoskeletal disease, mental health disease, and COPD as selected disease from the respiratory disease cluster.

The following diseases were included within the disease clusters of special interest (conditionally chronic disease are marked with an asterisk*):

Cardiovascular disease:

- Angina pectoris
- Atrial fibrillation / flutter
- Myocardial infarction
- (Congestive) heart failure
- Intermittent claudication
- TIA (transient ischemic attack)
- CVA (cerebrovascular accident)
- Heart valve disease
- Peripheral arterial disease / Raynaud’s disease*
- Chronic venous insufficiency / chronic varicosis*
- Congenital heart defects*
- Hypertension^†^

^†^In the current study, with SBP as one of the primary outcome measures, presence of hypertension as diagnostic label in the CMR registry had to be complemented with at least one other cardiovascular disease diagnostic label for patients to be analyzed longitudinally within the cardiovascular disease cluster.

Malignancy:

- Breast cancer
- Prostate cancer
- Endometrial cancer
- Skin cancer (only the types with potential to metastasize, e.g. basal cell carcinoma was excluded)
- Colon cancer
- Rectal cancer
- Lung / bronchial cancer
- Bladder cancer
- Ovarian cancer
- Uterine cervical cancer
- Brain cancer
- Leukemia
- Lymphoma / multiple myeloma
- Cancer of the stomach
- Esophageal cancer
- Pancreatic cancer
- Cancer of the mouth / pharynx
- Laryngeal / throat cancer
- Other specified types of cancer or metastases of unknown origin

Musculoskeletal disease:

- Rheumatoid arthritis; ankylosing spondylarthritis
- Osteoarthritis of the knee
- Osteoarthritis of the hip
- Osteoarthritis of the lumbar or cervical spine
- Other specified osteoarthritis
- Osteoporosis
- Polymyalgia rheumatica; giant cell arteritis*

Mental health disease:

- Depression*
- Anxiety disorder*
- Personality disorder
- Obsessive-compulsive disorder*
- Schizophrenia
- Psychosis*
- Phobia*
- (Chronic) functional somatic symptoms*
- Alzheimer’s disease
- Mental retardation

Respiratory disease:

As fifth separate disease group of special interest, COPD as a single disease was analyzed for associations with the longitudinal study outcomes, since within the cluster of respiratory diseases, COPD constituted the large majority of diseases (79%), and with that its prevalence was large enough to be studied on itself. This ensured good homogeneity within this comorbidity group, since COPD has a distinct therapeutic approach from the other diseases. The following diseases were considered within the entire cluster of respiratory diseases and could contribute to the total number of comorbid diseases:

- COPD (chronic obstructive pulmonary disease)
- Sarcoidosis*
- Asthma*
- Pneumoconiosis
- Bronchiectasis
- Chronic sinusitis*

**References**

1 Luijks H, Schermer T, Bor H, van Weel C, Lagro-Janssen T, Biermans M*, et al.* Prevalence and incidence density rates of chronic comorbidity in type 2 diabetes patients: an exploratory cohort study. *BMC Med* 2012; **10**: 128.
